# No evidence for hypogammaglobulinemia in patients with paroxysmal nocturnal hemoglobinuria (PNH) chronically treated with ravulizumab

**DOI:** 10.1371/journal.pone.0230869

**Published:** 2020-03-27

**Authors:** Ferras Alashkar, Scott Rottinghaus, Colin Vance, Dörte Herich-Terhürne, Ulrich Dührsen, Roland Assert, Alexander Röth

**Affiliations:** 1 Department of Hematology, West German Cancer Center, University Hospital Essen, Essen, Germany; 2 Alexion Pharmaceuticals, Inc, Boston, MA, United States of America; 3 Rheinisch-Westfälisches Institut für Wirtschaftsforschung, Essen, Germany; 4 Department of Clinical Chemistry, University Hospital Essen, Essen, Germany; Mayo Clinic, UNITED STATES

## Abstract

**Introduction:**

Ravulizumab (ALXN1210) is a long-lasting recycling IgG monoclonal antibody with an increased affinity for the neonatal Fc receptor (FcRn). The FcRn is essential for regulating IgG homeostasis. Saturation of the FcRn pathway is seen under high IgG doses as they compete with endogenous IgG to bind the FcRn by their Fc regions, resulting in enhanced IgG clearance.

**Patients/Methods:**

Between Jan 2016 and Jun 2019 (median observation time 21.6 months (6–37.7 months)) serum IgG concentrations and IgG1-4 subclasses were evaluated over a longitudinal course (post-hoc analysis) in 12 ravulizumab-treated adult patients with paroxysmal nocturnal hemoglobinuria (PNH) (58% (7/12) males, median age 50 years (yrs) (18–70 yrs)). All patients were enrolled in one of the three ravulizumab-PNH-related trials (201-, 301-, or 302-study) at the University Hospital Essen.

**Results:**

Baseline IgG concentrations were documented in 11 out of the 12 patients prior to ravulizumab treatment (median IgG 9.9 g/L (5–13.5 g/L)). In two female patients a clinically not relevant hypogammaglobulinemia with an associated IgG1 or a combined IgG1/IgG2 deficiency prior to treatment was documented. The data were further stratified with regard to various treatment intervals as multiple analyses were obtained. Throughout observation time IgG concentrations remained within physiologic ranges with no evidence of a treatment-related IgG depletion (median IgG at study endpoint 10.1 g/L (6–13.4 g/L)).

**Conclusion:**

In ravulizumab-treated PNH patients, IgG and IgG subclass levels which are regulated by the FcRn remained unaffected. Therefore, no treatment associated hypogammaglobulinemia is to be feared under chronic ravulizumab therapy.

## Introduction

Paroxysmal nocturnal hemoglobinuria (PNH) is a chronic and debilitating disorder characterized by chronic and uncontrolled dysregulation of the alternative complement pathway with complement-mediated intravascular and to minor extent extravascular hemolysis. Patients experience severe thrombophilia with an increased risk for both arterial and venous thromboses even at atypical sites, and various degrees of cytopenia—related either to ongoing hemolysis or bone marrow failure or due to a combination of both. By its nature, PNH is a clonal disorder affecting either one or several pluripotent hematopoietic stem cells of the bone marrow harboring a genetically acquired somatic loss of function mutations in the phosphatidylinositol N-acetylglucosaminyltransferase subunit A (PIG-A) which is encoded by the PIGA gene resulting in a defective glycosylphosphatidylinositol (GPI)-anchored protein synthesis with a reduced expression or even absence of GPI-anchored proteins, in particular CD55 and CD59. CD55- and CD59-deficient PNH erythrocytes are vulnerable to complement-mediated hemolysis secondary to increased activity of C3 convertases, as CD55 inhibits C3 convertases, whereas CD59 via its inhibitory role of C9 incorporation into the terminal complement complex, blocks the formation of the membrane attack complex (MAC) [1–3]. Of note, the exact mechanism for clonal expansion remains unclear, presuming an immune-mediated (secondary) selective advantage of PNH clones over normal hematopoietic cells [4].

The devastating nature of PNH demands timely diagnosis and intervention. In PNH, effective treatment is based on terminal complement modulation, with C5 inhibition reflecting the mainstay in PNH treatment. Since June 2007 the recombinant humanized monoclonal C5-antibody (mAb) eculizumab has been approved in the EU for the treatment of symptomatic PNH patients and is considered to this date the standard of care, resulting in a rapid and dramatic improvement of PNH-related symptoms and complications, improving overall survival (OS) and quality of life (QoL) [5–9]. Eculizumab prevents complement-dependent intravascular hemolysis via inhibition of terminal complement activation by selective binding to C5, thereby inhibiting the subsequent generation of the pro-inflammatory, prothrombotic complement fragments C5a and C5b, thereby preventing downstream formation of the MAC while preserving upstream C3-mediated activity, which is required for clearance of micro-organisms and immune complexes [10].

Ravulizumab (ALXN1210), a second generation anti-C5 mAb engineered from eculizumab, has recently received marketing authorization for the treatment of adult patients with PNH in the United States, EU, and Japan, based on the positive results of the two published Phase 3 ALXN1210-301 and 302 clinical trials demonstrating an equal efficacy and a similar safety profile to the current standard of care treatment with eculizumab [11–13]. Ravulizumab provides highly specific C5 inhibition. Moreover, it is recycled more effectively than eculizumab through the neonatal Fc receptor (FcRn) pathway due to its increased affinity for the FcRn and rapid endosomal dissociation of the ravulizumab-C5 complex, allowing lysosomal degradation of C5 while recycling ravulizumab to the vascular space through the FcRn. This results in an extended half-life allowing an 8 week dosing interval for ravulizumab compared to 2 weeks for eculizumab. Ravulizumab provides rapid and sustained reduction in complement-mediated hemolysis, while effectively reducing the rate of breakthrough hemolysis which is seen in up to 27% in eculizumab-treated patients [14–17].

## Primary objective

The primary objective of this monocentric, post-hoc analysis performed at the University Hospital of Essen was designed to evaluate whether or not in ravulizumab-treated adult PNH patients ≥ 18 years (yrs) of age endogenous IgG depletion might be observed under chronic treatment with ravulizumab. This hypothesis was generated after an 18-year-old male patient with no evidence of an associated IgG deficiency prior to treatment with ravulizumab experienced a penicillin-resistant serogroup Y meningococcal infection. Thus, we speculated that endogenous IgG depletion might be feared in patients receiving ravulizumab, secondary to hypercatabolism of IgG either via FcRn saturation and/or competition for the FcRn-binding site, consequently increasing the risk for serious infectious complications in patients receiving ravulizumab.

## Patients and methods

In this single‐center, post-hoc analysis, IgG serum concentrations and the associated IgG1, IgG2, IgG3, and IgG4 subclasses were assessed over a longitudinal course following treatment with ravulizumab at the Central Laboratory at the University Hospital of Essen (DIN EN ISO 15189) by nephelometry (Dimension Vista^®^ 1500, Siemens Healthineers) in 12 ravulizumab-treated PNH patients ≥ 18 years of age over time (reference values: IgG 7–16 g/L; IgG1 4050–10110 mg/L, IgG2 1690–7860 mg/L, IgG3 110–850 mg/L, IgG4 30–2010 mg/L). Patients’ clinical characteristics by the time of IgG serum and IgG1-4 subclass assessment are shown in Table 1.

**Table 1:** Baseline clinical characteristics.

Number of patients	12
Median age in yrs (range)	50 (18–70)
Gender, male / female (%)	7 (58) / 5 (42)
Peripheral blood counts	
• Hb (g/dL), median (range)	11.258.8–14.2)
• WBC (/nL), median (range)	3.9 (2.1–4.9)
• Plts (/nL), median (range)	154.5 (64–185)
• LDH (U/L), median (range)	252 (184–386)
PNH clone, median (range), n (%) (Gran. FLAER)	73.1 (36–99)
Albumin (g/dL)	4.6 (4.2–4.9)
Total protein (g/dL)	6.91 (6.3–7.7)

**Abbreviations:** ARC, absolute reticulocyte count; Hb, hemoglobin; FLAER, fluorescein-labeled proaerolysin; Gran., granulocyte; LDH, lactate dehydrogenase; Plts, platelets; PNH, paroxysmal nocturnal hemoglobinuria; WBC, white blood count; yrs, years.

Albumin concentrations were measured on the basis of the method of Doumas and Biggs which contains bromocresol green as a binding dye for albumin[18]. Total protein (TP) concentrations were determined based on the method of Weichselbaum using biuret reagent with cupric sulfate which builds cuproproteinate complexes in an alkaline solution [19]. Lactate Dehydrogenase L-P (LDH) activity was measured by its conversion of L-lactate to pyruvate in the presence of nicotinamide adenine dinucleotide based on the method of Amador et al. [20]. In serum albumin concentrations, TP concentrations and LDH activity were measured with an automated clinical chemistry analyzer (Advia® 2400 Chemistry System, Siemens Healthcare, Erlangen, Germany). For white blood cell and platelet counts we used the Sysmex XN-1000 hematology analyzer. Hemoglobin concentrations were measured with the SLS method of Sysmex with the same analyzer.

These 12 patients were enrolled in the phase 2, open-label, non-randomized ALXN1210-PNH-201 study (4 patients) or the two open-label, randomized, active-controlled, non-inferiority phase 3 studies: ALXN1210-PNH-301 (2 patients) and ALXN1210-302 (6 patients) at the University Hospital of Essen. The ALXN1210-PNH-201 study was designed to evaluate the safety, tolerability, and efficacy of multiple intravenous doses of ravulizumab administered to complement inhibitor treatment-naïve adult PNH patients ≥ 18 years of age, whereas the ALXN1210-PNH-301 and ALXN1210-PNH-302 studies were evaluating the non-inferiority of ravulizumab versus open-label eculizumab in either treatment-naïve (study 301) or in clinically stable adult PNH patients ≥ 18 years of age previously treated with open-label eculizumab for a minimum period of at least 6 months [11], [12], [21].

Typing of suspected monoclonal immunoglobulins was done by serum protein electrophoresis followed by immunofixation with specific antibodies (HYDRAGEL® IF, SEBIA, Evry, France).

Before study participation, all patients provided written informed consent. Retrospective analysis and use of data was approved by the Ethical Committee of the Faculty of Medicine at the University Hospital of Duisburg-Essen. The study was conducted in accordance to the Declaration of Helsinki.

The ALXN1210-PNH-201, ALXN1210-PNH-301 and ALXN1210-302 studies were conducted in accordance with the Declaration of Helsinki and the International Council for Harmonization Good Clinical Practice Guidelines. These trials were registered at www.clinicaltrials.gov as NCT02605993 (study 201), NCT02946463 (study 301), and NCT03056040 (study 302).

## Results

**Fig 1:**
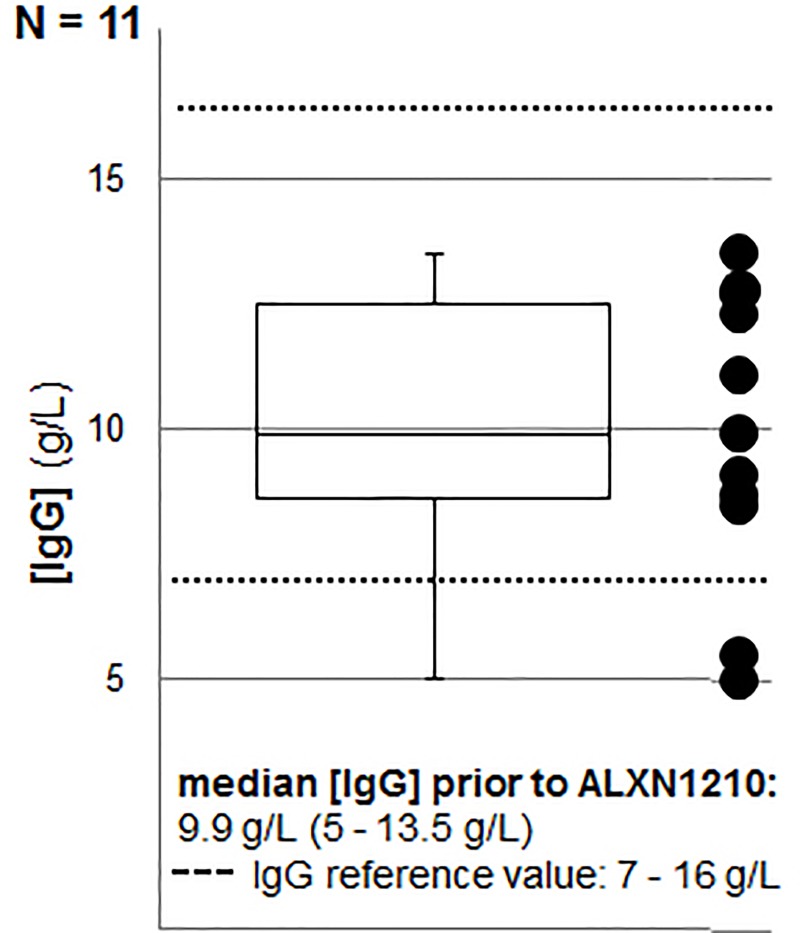
IgG concentrations at baseline in adult PNH patients prior to enrollment in the ALXN1210 studies (n = 11).

In the 12 adult PNH patients median observation time following treatment initiation with ravulizumab was 21.6 months (6–37.7 months). In one male patient IgG concentrations prior to treatment were not available at baseline, as study enrollment was in Canada prior to transfer to our department for treatment continuation due to transfer of residency. The corresponding baseline IgG concentrations in the remaining eleven patients are depicted in Fig 1, excluding a pre-existing hypogammaglobulinemia in nine of the eleven patients at baseline, as IgG concentrations were within the physiologic ranges of 7–16 g/L (median 10.5 g/L; 8.5–13.5 g/L). An IgG-related hypogammaglobulinemia was evident in two female patients prior to study enrollment in the ALXN1210-PNH-301 and 302 trials (patient 1: 25 years (yrs) of age; IgG 5.5 g/L, IgG1 3360 mg/L (day 382); patient 2: 50 yrs of age; IgG 5 g/L, IgG1 3680 mg/L, IgG2 229 mg/L (day 231)).

**Fig 2:**
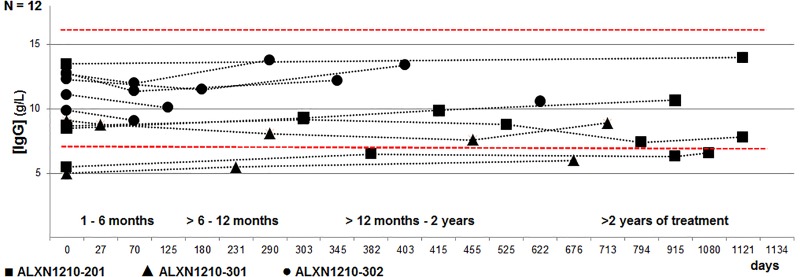
Longitudinal course of IgG concentrations under treatment with ravulizumab (n = 12).

In Fig 2, a longitudinal distribution of the IgG concentrations measured over time in the 12 patients receiving ravulizumab with further differentiation according to the enrolled study is shown. In the 11 patients receiving ravulizumab in whom baseline IgG concentrations were obtained, IgG levels remained within the normal ranges without evidence of a secondary, ravulizumab-associated IgG depletion throughout the observation time. Of further interest was a 20% increase in IgG concentrations during ravulizumab treatment in the two female patients with an IgG deficiency prior to treatment (patient 1: IgG 5.5 g/L at baseline, IgG 6.6 g/L at the endpoint of observation; patient 2: IgG 5 g/L at baseline, IgG 6 g/L at the endpoint of observation).

As previously mentioned, four patients (male to female ratio: 1:1, median IgG follow-up time 36.7 months (30.5–37.8 months), median age 35.5 yrs (18–51 yrs) by the time of study enrollment) participated in the multiple ascending dose ALXN1210-PNH-201 study (cohort 1: two patients, cohort 2: one patient, cohort 3: one patient). In these patients, dosing of ravulizumab—according to the currently approved weight-dependent dosing recommendation for ravulizumab—was administered for a median of 16.6 months (range 15.2–17.6 months). IgG concentrations in these patients, despite dose modifications according to the ALXN1210-201 study, did not result in any changes in IgG concentrations following ≥ 2 years of treatment (median IgG concentrations at baseline: 8.6 g/L (range 5.5–13.5 g/L); ≥ 2 yrs following treatment with ALXN1210: median 9.1 g/L (range 6.6–14 g/L)).

In the eight patients enrolled in the ALXN1210-301 and -302 trials (62% males (5/8), median age 55.5 yrs (22–70 yrs) by the time of study enrollment), median follow-up starting from treatment initiation with ravulizumab was 12.5 months (range 6–27.8 months). For further completeness, we report results on four patients (50%) who were either randomized to the ALXN1210-301 eculizumab control-arm, or received eculizumab prior to trial enrollment. Baseline IgG concentrations were documented in seven out of these eight patients. Despite the fact that IgG concentrations in the one male patient due to change of residency were not obtained at baseline, in this patient no IgG depletion was observed throughout follow-up, with physiologic values being initially documented following 20.7 months of treatment with ravulizumab in the 302-study. Therefore, in consideration of the obtained data, a preexisting hypogammaglobulinemia might further be excluded.

Given the overall small sample size in addition to baseline IgG concentrations (n = 11), we further stratified the obtained data with determination of IgG concentrations with regard to different treatment intervals following initiation with ravulizumab (months 1 - < 6 (6 patients), ≥ 6 - < 12 months (7 patients), ≥ 12 months—< 2 yrs (6 patients), and ≥ 2 yrs of treatment (4 patients)), as multiple analyses were obtained in each patient throughout observation time, resulting in a more representative correlation over time and under treatment (Fig 2). Following initiation of ravulizumb, IgG concentrations remained stable with no evidence of IgG depletion in patients with no prior evidence of an IgG deficiency at baseline over time.

**Fig 3:**
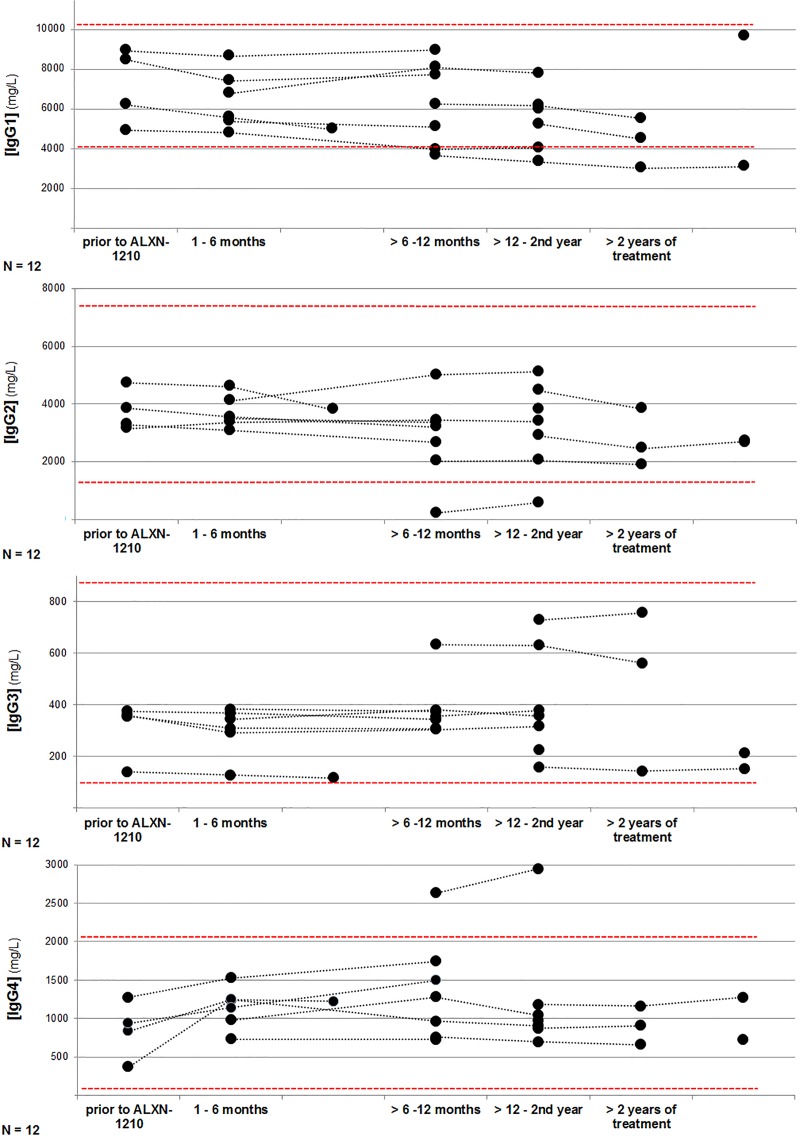
Longitudinal course of IgG subclass (IgG1, IgG2, IgG3, and IgG4) concentrations under treatment with ravulizumab (n = 12).

In the second step, we evaluated the longitudinal course of IgG subclasses (IgG1, IgG2, IgG3, and IgG4) in these patients under treatment with ravulizumab (Fig 3). At baseline, associated IgG subclasses were obtained in four patients (62.5 yrs by the time of study enrollment, median observation time 8.8 months). In these four patients IgG subclasses were within the physiological ranges prior to treatment and remained stable during follow-up with no evidence of an associated IgG subclass depletion.

In the remaining eight patients, IgG subclasses were evaluated throughout treatment period at a median observation time of 10.1 months (range 2.3–37.4 months), with 62.5% of the patients (5/8) being evaluated at a minimum time interval of 12 months post treatment initiation. Even though IgG subclasses were not routinely determined at baseline in the cohort population and therefore an IgG subclass deficiency could in general not be excluded prior to treatment, no variations in the corresponding IgG subclass levels over time were observed. Therefore, when correlating these data with baseline IgG levels, physiologic IgG subclass concentrations might be assumed.

As previously stated, in the two female patients with an documented IgG deficiency at baseline, an associated IgG1 deficiency was subsequently diagnosed in one of them (patient 1: IgG1 3360 mg/L (day 382 of treatment)), whereas in the other patient a combined IgG1 and IgG2 deficiency was evident (patient 2: IgG1 3680 mg/L, IgG2 229 mg/L (day 231 of treatment)). However, in the female patient with a combined IgG subclass deficiency a concomitant increase in IgG4 levels was noticed under treatment with ravulizumab. The longitudinal course of IgG subclasses are provided in Fig 3, providing further evidence that no treatment-related IgG subclass depletion was observed in these patients over time. This especially accounts for patients in whom the corresponding values prior to treatment were obtained.

Due to the small sample size, an age-related stratification was omitted.

Of note, in 10 patients following treatment with ravulizumab, a faint monoclonal IgG kappa band identified by serum protein electrophoresis and immunofixation became apparent in ravulizumab-treated patients over time.

## Discussion

Ravulizumab (ALXN1210) has recently been approved in the United States and Europe for the treatment of adult patients ≥ 18 years of age with symptomatic PNH. The approval provided by the European Commission was based on comprehensive results from the two phase 3 studies in either complement-naïve or eculizumab-pretreated adult patients. In these studies ravulizumab was non-inferior to standard of care treatment with eculizumab when administrated every 8 weeks, in contrast to the labeled-dose eculizumab given every 14±2 days in both complement-naïve (301-study) and eculizumab-pre-experienced adult patients (302-study). Furthermore, ravulizumab had a similar safety profile to eculizumab in both studies [11], [12].

Based on these data, in addition to prior results obtained in the two phase 1b/2 multicenter open-label 103- and 201-studies [21], ravulizumab might change the treatment paradigm in PNH patients, reducing treatment burden in adult patients with PNH.

The mAb ravulizumab is directed against complement component C5. Ravulizumab has a high binding affinity to complement protein C5, preventing cleavage to complement proteins C5a and C5b with downstream inhibition of terminal complement complex (C5b-9) generation, proving immediate and complete inhibition of complement protein C5 (free C5 levels of <0.5 mcg/mL). This C5 inhibition prevents complement-mediated inflammation and intravascular hemolysis while preserving early complement pathway activation, which is especially essential for clearance of immune complexes and microbial opsonization. Free C5 levels of <0.5 mcg/ml have been correlated with maximal intravascular hemolysis control and complete terminal complement inhibition [11], [12]. Therefore, despite high levels of circulating ravulizumab, saturation of the FcRn recycling pathway potentially leading to increased ravulizumab clearance might not need to be feared as complement inhibition provided by ravulizumab is sustained [11], [12], [17], [21].

Of further importance remains the fact, that complement C5a is known to modulate the balance between activating versus inhibitory IgG Fc receptors on leukocytes, thereby enhancing autoimmune responses in human, and cleavage of C5 is inhibited by eculizumab [22].

Eculizumab is an IgG kappa immunoglobulin with an engineered Fc portion which is a hybrid of IgG2 and IgG4 [23]. Ravulizumab differs from eculizumab in its structure by the substitution of four amino acids (Tyr-27-His, Ser-57-His, Met-429-Leu, and Asn-435-Ser) into the heavy chain of eculizumab and the neonatal Fc regions of the eculizumab backbone altering its pharmacokinetics and -dynamics with extension of its half-life up to approximately five-fold in contrast to eculizumab. Therefore, extending dosing to once every eight weeks is possible. Whereas, residues in the variable region of this mAb ensuring the affinity to directly bind to C5—as does eculizumab—were not altered. The histidine substitutions consequently 1) reduce ravulizumab-C5 complex clearance via an antigen-mediated clearance by enhancing lysosomal degradation of C5 as the dissociation of the ravulizumab-C5 complex in the endosome is attenuated and 2) increase the affinity and efficacy of the FcRn‐mediated ravulizumab recycling in the vascular space as this mAb demonstrates an increased affinity for the FcRn [11], [12], [21].

Structurally, the FcRn is a major histocompatibility complex (MHC) class 1‐related protein consisting of an α‐chain with three extracellular domains linked non‐covalently with β2‐microglobulin, and was first discovered in rats, where it was found to mediate the transportation of IgG from the mother’s milk across the newborn’s gut epithelium into its bloodstream, conferring passive humoral immunity [24–26]. In human, the FcRn is essential for maintaining and regulating IgG homeostasis by protecting IgG from lysosomal degradation via an endosomal re‐routing pathway with recycling of internalized IgG molecules back to the cell surface [27].

Clearance of IgG is enhanced following administration of high doses of IgG as the FcRn‐dependent regulation of IgG concentrations is a saturable process, whereas IgM, IgA and albumin concentrations remain unaffected. Furthermore, IgG catabolism is enhanced secondary to antibodies demonstrating an increased binding affinity for the FcRn due to competing with endogenous IgG for FcRn binding [28–30].

Taking this into account, in addition to the penicillin-resistant serogroup Y meningococcal infection in an 18-year old male patient receiving ravulizumab in the 201-study on day 222 of treatment with no evidence of a concomitant IgG deficiency prior to treatment, this analysis was designed to evaluate whether or not in ravulizumab-treated adult PNH patients, endogenous IgG depletion might be observed. Endogenous IgG depletion under chronic ravulizumab treatment might be speculated to result secondary to hypercatabolism of IgG either via FcRn saturation and/or competition for the FcRn-binding site, which consequently increases the risk for infectious complications due to IgG deficiency. Of note, this patient did not receive a penicillin prophylaxis throughout treatment according to local policy. However, he had been vaccinated against meningococcal serogroups A, C, W, and Y and serogroup B in accordance to recommendations for patients receiving eculizumab and the ALXN1210-PNH-201 study protocol.

The results of this study suggest that the hypothesis of a profound IgG or an associated IgG subclass hypercatabolism cannot be supported, as alternations in endogenous IgG concentrations resulting in hypogammaglobulinemia secondary to ravulizumab were not observed. In the two patients with a prior to treatment documented IgG deficiency, IgG or IgG subclass concentrations were not affected throughout the observation time. Furthermore, in these two patients there was no evidence of an increased susceptibility for infectious complications under treatment with ravulizumab over time.

An increase in IgG4 subclass levels in addition to evidence of a faint monoclonal IgG kappa band in the serum protein electrophoresis and immunofixation following ravulizumab administration was explained by the fact that ravulizumab, which is engineered from eculizumab, is an IgG 2/4 kappa mAb, causing a faint monoclonal band for IgG kappa in the serum protein electrophoresis and immunofixation, similar to patients treated with rituximab, and therefore was considered to reflect circulating ravulizumab, further providing evidence of the long half-life of ravulizumab [31]. Furthermore, a positive immunofixation has previously been reported in eculizumab-treated patients [32].

Since high doses of IgG result in saturation of the FcRn pathway, careful co-administration of IgG replacement therapy in PNH patients with co-existing medical conditions (e.g. immune thrombocytopenic purpura (ITP), clinically relevant hypogammaglobulinemia, multiple sclerosis) is recommended as this might further result in enhanced clearance of ravulizumab, as intravenous IgG preparations compete with endogenous IgG to bind the FcRn by their Fc regions. This in turn might increase the risk for breakthrough hemolytic events in ravulizumab-treated PNH patients, and might shorten the treatment interval, necessitating an additional ravulizumab dose following IgG replacement therapy. However, mAbs administrated at a dose of ~0.5 to 10 mg/kg or endogenous IgG levels are not expected to saturate the FcRn pathway [33], whereas blockage of the FcRn by genetically engineered antibodies in order to achieve a rapid decrease in total IgG and autoantibodies can be achieved at doses of 4‐20 mg/kg [34].

In summary, the results obtained in this retrospective, monocentric post-hoc analysis are the first data in ravulizumab-treated adult PNH patients investigating IgG concentrations over a longitudinal course, showing that IgG and IgG subclass levels which are regulated by the FcRn remain unaffected. Therefore, in these patients the fear of a treatment-related hypogammaglobulinemia resulting in an increased susceptibility for infectious complications and furthermore re‐occurrence of PNH‐related symptoms or breakthrough hemolysis can be abandoned.

## Abbreviations

FcRn: neonatal Fc receptor
GPI: glycosylphosphatidylinositol
Ig: immunoglobulin
ITP: immune thrombocytopenic purpura
LDH: lactate Dehydrogenase
mAb: monoclonal antibody
MAC: membrane attack complex
MHC: major histocompatibility complex
PIG-A: phosphatidylinositol N-acetylglucosaminyltransferase subunit A
OS: overall survival
PNH: paroxysmal nocturnal hemoglobinuria
TP: total protein
QoL: quality of life
yrs: years

## References

[1] Risitano A. M. and Rotoli B., “Paroxysmal nocturnal hemoglobinuria: pathophysiology, natural history and treatment options in the era of biological agents.,” *Biologics*, vol. 2, no. 2, pp. 205–22, 6 2008 10.2147/btt.s1420 19707355 PMC2721357

[2] Hill A., DeZern A. E., Kinoshita T., and Brodsky R. A., “Paroxysmal nocturnal haemoglobinuria.,” *Nat*. *Rev*. *Dis*. *Prim*., vol. 3, no. 1, p. 17028, 12 2017. 28516949 10.1038/nrdp.2017.28 PMC7879566

[3] Brodsky R. A., Ginsburg D., Smith B. R., Nathan D. G., Orkin S. H., and Rappeport J. M., “Paroxysmal nocturnal hemoglobinuria.,” *Blood*, vol. 124, no. 18, pp. 2804–11, 10 2014 10.1182/blood-2014-02-522128 25237200 PMC4215311

[4] Lee S. C.-W. and Abdel-Wahab O., “The mutational landscape of paroxysmal nocturnal hemoglobinuria revealed: new insights into clonal dominance.,” *J*. *Clin*. *Invest*., vol. 124, no. 10, pp. 4227–30, 2014 10.1172/JCI77984 25244089 PMC4191026

[5] Hillmen P. et al., “Effect of the complement inhibitor eculizumab on thromboembolism in patients with paroxysmal nocturnal hemoglobinuria,” *Blood*, vol. 110, no. 12, pp. 4123–4128, 12 2007 10.1182/blood-2007-06-095646 17702897

[6] Hillmen P. et al., “Effect of eculizumab on hemolysis and transfusion requirements in patients with paroxysmal nocturnal hemoglobinuria.,” *N*. *Engl*. *J*. *Med*., vol. 350, no. 6, pp. 552–9, 2 2004 10.1056/NEJMoa031688 14762182

[7] Brodsky R. A. et al., “Multicenter phase 3 study of the complement inhibitor eculizumab for the treatment of patients with paroxysmal nocturnal hemoglobinuria.,” *Blood*, vol. 111, no. 4, pp. 1840–7, 2 2008 10.1182/blood-2007-06-094136 18055865

[8] Hillmen P. et al., “The Complement Inhibitor Eculizumab in Paroxysmal Nocturnal Hemoglobinuria,” *N*. *Engl*. *J*. *Med*., vol. 355, no. 12, pp. 1233–1243, 9 2006 10.1056/NEJMoa061648 16990386

[9] Loschi M. et al., “Impact of eculizumab treatment on paroxysmal nocturnal hemoglobinuria: a treatment versus no-treatment study,” *Am*. *J*. *Hematol*., vol. 91, no. 4, pp. 366–370, 6 2016 10.1002/ajh.24278 26689746

[10] Hill A. et al., “Eculizumab prevents intravascular hemolysis in patients with paroxysmal nocturnal hemoglobinuria and unmasks low-level extravascular hemolysis occurring through C3 opsonization.,” *Haematologica*, vol. 95, no. 4, pp. 567–73, 4 2010 10.3324/haematol.2009.007229 20145265 PMC2857185

[11] Kulasekararaj A. G. et al., “Ravulizumab (ALXN1210) vs eculizumab in C5-inhibitor–experienced adult patients with PNH: the 302 study,” *Blood*, vol. 133, no. 6, pp. 540–549, 2 2019 10.1182/blood-2018-09-876805 30510079 PMC6368201

[12] Lee J. W. et al., “Ravulizumab (ALXN1210) vs eculizumab in adult patients with PNH naive to complement inhibitors: the 301 study,” *Blood*, vol. 133, no. 6, pp. 530–539, 2 2019 10.1182/blood-2018-09-876136 30510080 PMC6367644

[13] CHMP, “Committee for Medicinal Products for Human Use (CHMP),” 2019.

[14] Peffault de Latour R. et al., “Assessing complement blockade in patients with paroxysmal nocturnal hemoglobinuria receiving eculizumab.,” *Blood*, vol. 125, no. 5, pp. 775–83, 1 2015 10.1182/blood-2014-03-560540 25477495

[15] Nakayama H., Usuki K., Echizen H., Ogawa R., and Orii T., “Eculizumab Dosing Intervals Longer than 17 Days May Be Associated with Greater Risk of Breakthrough Hemolysis in Patients with Paroxysmal Nocturnal Hemoglobinuria,” *Biol*. *Pharm*. *Bull*., vol. 39, no. 2, pp. 285–288, 2016 10.1248/bpb.b15-00703 26830487

[16] Hillmen P. et al., “Long-term safety and efficacy of sustained eculizumab treatment in patients with paroxysmal nocturnal haemoglobinuria.,” *Br*. *J*. *Haematol*., vol. 162, no. 1, pp. 62–73, 7 2013 10.1111/bjh.12347 23617322 PMC3744747

[17] Sheridan D. et al., “Design and preclinical characterization of ALXN1210: A novel anti-C5 antibody with extended duration of action,” *PLoS One*, vol. 13, no. 4, p. e0195909, 4 2018 10.1371/journal.pone.0195909 29649283 PMC5897016

[18] DOUMAS B. T., BIGGS H. G., ARENDS R. L., and PINTO P. V. C., “Determination of Serum Albumin,” *Stand*. *Methods Clin*. *Chem*., vol. 7, pp. 175–188, 1 1972.

[19] WEICHSELBAUM T. E., “An accurate and rapid method for the determination of proteins in small amounts of blood serum and plasma.,” Am. J. Clin. Pathol., vol. 10, pp. 40–49, 3 1946 21027099

[20] AMADOR E., DORFMAN L. E., and WACKER W. E., “SERUM LACTIC DEHYDROGENASE ACTIVITY: AN ANALYTICAL ASSESSMENT OF CURRENT ASSAYS.,” *Clin*. *Chem*., vol. 12, pp. 391–9, 8 1963 14060190

[21] Röth A. et al., “Ravulizumab (ALXN1210) in patients with paroxysmal nocturnal hemoglobinuria: results of 2 phase 1b/2 studies.,” *Blood Adv*., vol. 2, no. 17, pp. 2176–2185, 2018 10.1182/bloodadvances.2018020644 30171081 PMC6134221

[22] Manthey H. D., Woodruff T. M., Taylor S. M., and Monk P. N., “Complement component 5a (C5a),” *Int*. *J*. *Biochem*. *Cell Biol*., vol. 41, no. 11, pp. 2114–2117, 11 2009 10.1016/j.biocel.2009.04.005 19464229

[23] Hillmen P., “The role of complement inhibition in PNH.,” *Hematol*. *Am*. *Soc*. *Hematol*. *Educ*. *Progr*., vol. 2008, no. 1, pp. 116–23, 1 2008. 10.1182/asheducation-2008.1.116 19074068

[24] Simister N. E. and Rees A. R., “Isolation and characterization of an Fc receptor from neonatal rat small intestine,” *Eur*. *J*. *Immunol*., vol. 15, no. 7, pp. 733–738, 1 1985 10.1002/eji.1830150718 2988974

[25] Brambell F. W., “The transmission of immunity from mother to young and the catabolism of immunoglobulins.,” *Lancet (London, England)*, vol. 2, no. 7473, pp. 1087–93, 11 1966. 10.1016/s0140-6736(66)92190-8 4162525

[26] Pyzik M., Rath T., Lencer W. I., Baker K., and Blumberg R. S., “FcRn: The Architect Behind the Immune and Nonimmune Functions of IgG and Albumin.,” *J*. *Immunol*., vol. 194, no. 10, pp. 4595–603, 5 2015 10.4049/jimmunol.1403014 25934922 PMC4451002

[27] Roopenian D. C. et al., “The MHC Class I-Like IgG Receptor Controls Perinatal IgG Transport, IgG Homeostasis, and Fate of IgG-Fc-Coupled Drugs,” *J*. *Immunol*., vol. 170, no. 7, pp. 3528–3533, 4 2003 10.4049/jimmunol.170.7.3528 12646614

[28] R. Kontermann, *Therapeutic proteins: strategies to modulate their plasma half-lives*.

[29] FAHEY J. L. and ROBINSON A. G., “FACTORS CONTROLLING SERUM GAMMA-GLOBULIN CONCENTRATION.,” *J*. *Exp*. *Med*., vol. 118, no. 5, pp. 845–68, 11 1963. 14087625 10.1084/jem.118.5.845 PMC2137682

[30] Pyzik M., Sand K. M. K., Hubbard J. J., Andersen J. T., Sandlie I., and Blumberg R. S., “The Neonatal Fc Receptor (FcRn): A Misnomer?,” *Front*. *Immunol.*, vol. 10, 7 2019. 10.3389/fimmu.2019.01540 PMC6636548 31354709

[31] Guancial E. A., Mahajan V. S., McCaffrey R. P., and Lindeman N., “Theraputic Monoclonal Antibody Interference In Immunofixation Electrophoresis,” *Blood*, vol. 116, no. 21, 2010.

[32] Röth A., Hock C., Konik A., Christoph S., and Dührsen U., “Chronic treatment of paroxysmal nocturnal hemoglobinuria patients with eculizumab: safety, efficacy, and unexpected laboratory phenomena,” *Int*. *J*. *Hematol*., vol. 93, no. 6, pp. 704–714, 6 2011 10.1007/s12185-011-0867-y 21611719

[33] A. S. Faqi, *A comprehensive guide to toxicology in nonclinical drug development*.

[34] Baldwin W. M., Valujskikh A., and Fairchild R. L., “The neonatal Fc receptor: Key to homeostasic control of IgG and IgG‐related biopharmaceuticals,” *Am*. *J*. *Transplant*., vol. 19, no. 7, p. ajt.15366, 4 2019. 10.1111/ajt.15366 PMC6591018 30903736

